# Dataset on ecological health and microbial communities of coastal aquaculture ponds from surrounding region of Sundarban mangroves

**DOI:** 10.1016/j.dib.2026.112542

**Published:** 2026-01-31

**Authors:** Yash, Anwesha Ghosh, Ajanta Dey, Milon Sinha, Nimai Bera, Sabyasachi Chakraborty, Punyasloke Bhadury

**Affiliations:** a Integrative Taxonomy and Microbial Ecology Research Group, Department of Biological Sciences, Indian Institute of Science Education and Research Kolkata, Mohanpur 741246 Nadia, West Bengal, India; b Centre for Climate and Environmental Studies, Indian Institute of Science Education and Research Kolkata, Mohanpur 741246 Nadia, West Bengal, India; c Nature Environment and Wildlife Society, 10 Chowringhee Terrace, Kolkata 700020 West Bengal, India

**Keywords:** Integrated mangrove aquaculture (IMA), Sustainable aquaculture in mangrove ecosystem fisheries (SAIME), Biological communities, eDNA, Nanopore, ICP-MS

## Abstract

Integrated Mangrove Aquaculture (IMA) and Sustainable Aquaculture in Mangrove Ecosystem Fisheries (SAIME) are key activities undertaken across coastal regions globally to meet growing demand for brackish-water aquaculture products through sustainable practices. An in-depth biomonitoring study was conducted to map the ecological health of IMA and non-IMA aquaculture ponds in the surrounding region of the Indian Sundarbans mangroves located along the northeast coast of Bay of Bengal. Surface water samples were collected from six aquaculture ponds, four IMA (IMA_C1, IMA_C3, IMA_DB1, and IMA_DB4) and two non-IMA (C6_NM and DB5_NM) in the month of October 2022, for characterizing niche-specific biological communities using the environmental DNA (eDNA) approach. During sampling, *in-situ* environmental parameters were recorded. Mangrove litter-derived phenolics (tannic and gallic acids) and dissolved nutrients were estimated using a UV–Vis spectrophotometer, while dissolved organic carbon (DOC) was measured with the elemental analyzer. Metal and metalloid concentrations were determined by inductively coupled plasma mass spectrometry approach (ICP–MS). IMA ponds showed ideal conditions for shrimp aquaculture, with pH ranging from 7.913 to 8.633 and dissolved oxygen (DO) between 5.32 and 6.03 mg/L, indicating no hypoxic conditions despite higher concentrations of phenolics. High-throughput sequencing (HTS) based on Oxford Nanopore Technologies (ONT) sequencing chemistry was undertaken on the MinION platform, revealing the predominance of Proteobacteria among prokaryotes and Bacillariophyta as well as Chlorophyta among eukaryotes from extracted eDNA in each studied pond. Additionally, members of the family Cyprinidae were also detected, reflecting the biodiversity of fish population in these ponds. Functional gene profiling indicated signatures associated with nitrogen, phosphorus, sulphur, potassium and iron acquisition and metabolism, along with pathways related to aromatic compound degradation. Overall, dissolved nutrients, dissolved organic carbon (DOC), metal and metalloid ion concentrations as well as structure and functional profiles of biological communities provide a comprehensive basis for evaluating the ecological health of aquaculture ponds. This study generates important baseline information for long-term monitoring and represents the first eDNA-based high-throughput sequencing assessment of IMA and non-IMA aquaculture ponds from surface water in close proximity to the Sundarbans mangrove.

Specifications Table

**Table:** 

Subject	Earth & Environmental Sciences
Specific subject area	Environmental Sciences and Microbial Ecology
Type of data	Raw and analysed
Data collection	During sampling, *in-situ* environmental parameters were measured in triplicate using handheld digital probes with ATC configuration. Surface water samples (1 L) were collected in wide-mouth sterile amber bottles and fixed with buffered 4 % formalin for dissolved nutrient estimations. At the same time, an additional 1 L of surface water was collected from each pond in sterile white HDPE bottles and immediately fixed with molecular-grade absolute ethanol for the extraction of environmental DNA (eDNA) and elucidation of microbial community structure. For the estimation of dissolved organic carbon (DOC) and phenolics [tannic acid (TA) and gallic acid (GA)], 40 mL of filtered, unfixed water was collected in glass vials with open-top, pierceable caps and butyl rubber septa. In addition, a separate 50 mL sample was collected and fixed with 2 % Suprapur HNO₃ for metal and metalloids analysis with ICP-MS.
Data source location	Region - Minakha Block, Chaital, North 24 Parganas and Kultali Subdivision, Madhabpur, South 24 Parganas, West Bengal Country – India Latitude Longitude IMA_C1 (22°30′40.9 N 088°46′54.7 E) IMA_C3 (22°29′59.8 N 088°47′56.2 E) C6_NM (22°30′20.1 N 088°44′14 E) IMA_DB1 (22°03′38 N 088°34′8.6 E) IMA_DB4 (22°03′19.8 N 088°34′13.6 E) DB5_NM (22°03′21.8 N 088°33′8.2 E)
Data accessibility	Repository name: SRA of NCBI Data identification number: SAMN51230763, SAMN51230764, SAMN51230765, SAMN51230766, SAMN51230767, SAMN51230768 Direct URL to data: https://www.ncbi.nlm.nih.gov/bioproject/PRJNA1321950

## 1 Value of the Data

• This is the first dataset generated from the coastal areas of the Sundarbans mangroves, providing baseline information on Integrated Mangrove Aquaculture (IMA), a sustainable practice adopted by aquaculture farmers to enhance bioresource yields.
• Generated dataset captures overall ecological health status of coastal aquaculture ponds by integrating multiple ecological parameters (*in-situ* physical, chemical, and biological dataset).
• Both IMA and non-IMA ponds (controls) are included to highlight ecological role of mangroves in coastal aquaculture ponds and their diverse positive impacts.
• The eDNA dataset of surface water, first from IMA ponds from the north east coastal Bay of Bengal and possibly from South Asia, serves as a biological proxy for assessing ecological health, particularly in relation to mangrove litter-driven nutrient stoichiometry and microbial niche-specific adaptations.
• This dataset also elucidates the structural and functional potential of microbial communities that are central to biogeochemical cycling in brackish water aquaculture systems. Notably, IMA ponds exhibited higher microbial richness (8 phyla and 42 families) compared to non-IMA ponds (6 phyla and 29 families), with enriched gene profiles linked to nitrogen, sulphur, aromatic compound metabolism, and carbon cycling, indicating improved ecological resilience and ecological stabilities with more balanced nutrient cycling capacity under the influence of integrated mangrove systems.
• This baseline dataset can serve as a valuable resource for defining the ecological health status of coastal aquaculture ponds. Integration of these data allows for the application of multiple ecological modelling approaches and computation of key indices such as the Water Quality Index (WQI), Water Production Index (WPI), Nemerow’s Pollution Index (NPI) and Trophic Level Index (TLI) to comprehensively assess overall ecosystem health.
• Further, information from this dataset can provide valuable insights for researchers, ecosystem managers, policymakers, and aquaculture practitioners to identify the carbon sequestration and biodiversity credit potential of IMA ponds as well as give a roadmap for ecological restoration in global coastal regions, while also supporting better livelihood opportunities for aquaculture-dependent coastal communities.

## 2 Background

Globally, aquaculture practices have increased multiple-fold in coastal regions due to rising market demand for aquaculture products, valued for their high nutritional content [1,2]. However, this rapid growth has come at a cost, resulting in the loss of 26.7 % of global mangroves due to coastal aquaculture practices, resulting in extensive landscape transformations across fragile coastal ecosystems [[3], [4], [5]]. Similar trends have been observed over the past few decades in the surrounding region of Sundarbans, the world’s largest contiguous mangrove wetland, an UNESCO World Heritage Site and a Ramsar Site, where unsustainable aquaculture practices have emerged as a challenge to biodiversity and ecosystem functioning [4,5]. For maximizing yields, excessive uses of feed and antibiotics have further impacted biodiversity in adjacent habitats. Integrated Mangrove Aquaculture (IMA), also referred to as Sustainable Aquaculture in Mangrove Ecosystem Fisheries (SAIME), represents a nature-based solution (NbS) that utilizes mangrove litter to promote sustainable aquaculture, restore ecosystem services, and improve the livelihoods of coastal aquaculture practitioners [[6], [7], [8]]. At the same time, IMA practices support the restoration and conservation of mangroves, providing long-term ecological and socio-economic benefits through the production of high market-demanding organic products. This study highlights the role of mangrove litter in four IMA ponds with integrated mangrove vegetation, compared to two non-IMA (control) ponds. Monitoring focused on mangrove litter-derived phenolic compounds (tannic and gallic acids), key environmental variables, biological communities and dissolved micro- and macronutrient dynamics [8]. Results showed that IMA ponds maintained a more balanced nutrient stoichiometry and supported higher bioresource yields compared to non-IMA ponds.

## 3 Data Description

The present study provides a comprehensive dataset offering an integrated ecological assessment of six coastal aquaculture ponds, including four Integrated Mangrove Aquaculture (IMA) ponds and two non-IMA ponds, located in the surrounding region of the Sundarbans, West Bengal, India. The dataset encompasses *in-situ* environmental parameters, along with estimations of dissolved nutrients, phenolic compounds, dissolved organic carbon (DOC) and quantifications of metal-metalloid ion concentrations for water quality evaluation of each studied pond. In parallel, taxonomic and functional profiles of prokaryotic and eukaryotic communities were elucidated from environmental DNA (eDNA) obtained from surface water of individual ponds under both models (IMA and non-IMA) using high-throughput sequencing (HTS) based on Oxford Nanopore Technologies (ONT) chemistry, conducted on the MinION platform. Collectively, these datasets enable a robust comparison between IMA and non-IMA systems and elucidate the influence of mangrove litter-mediated nutrient cycling on pond water quality, microbial community assemblages, their functional profiles and overall ecological health.

Environmental and geochemical parameters revealed clear distinctions between IMA and non-IMA ponds (Table 1, Table 2). DO concentrations were consistently higher in IMA ponds, reflecting well-oxygenated and metabolically active conditions favourable for brackish aquaculture and extensive shrimp aquaculture. In contrast, non-IMA ponds showed lower DO concentrations and higher DOC, indicating slower organic matter turnover and potential oxygen limitation. Nutrient concentrations, particularly nitrate and reactive silicate, were elevated in IMA ponds, supporting enhanced primary productivity and balanced nutrient cycling (Table 1).

**Table 1:** Environmental parameters were recorded from the surface water of four IMA (IMA_C1, IMA_C3, IMA_DB1, and IMA_DB4) and two non-IMA (C6_NM and DB5_NM) coastal aquaculture ponds located in North 24 Parganas and South 24 Parganas districts, West Bengal, India.

Environmental parameters	IMA_C1	IMA_C3	C6_NM	IMA_DB1	IMA_DB4	DB5_NM
Ambient temperature (AT) ( °C)	33.1 ± 0.00	35.5 ± 0.00	34.2 ± 0.00	33.0 ± 0.00	33.4 ± 0.00	36.4 ± 0.00
Surface water ( °C)	32.5 ± 0.00	34.1 ± 0.00	31.2 ± 0.00	30.2 ± 0.00	32.7 ± 0.00	32.3 ± 0.00
Dissolved Oxygen (DO) (mg/L)	5.32 ± 0.00	5.91 ± 0.00	5.12 ± 0.00	6.03 ± 0.02	5.16 ± 0.01	5.50 ± 0.03
pH	7.913 ± 0.00	8.132 ± 0.00	8.146 ± 0.00	8.633 ± 0.01	8.653 ± 0.01	8.512 ± 0.02
Total Dissolved Solids (TDS;ppm)	929 ± 0.00	948 ± 0.00	1378 ± 0.00	582 ± 0.00	317 ± 0.00	851 ± 0.00
Electrical Conductivity (EC;µS/cm)	1858 ± 0.00	1896 ± 0.00	2756 ± 0.00	1165 ± 0.00	632 ± 0.00	1708 ± 0.00
Secchi Depth (cm)	24 ± 0.00	27 ± 0.00	21 ± 0.00	24 ± 0.00	11 ± 0.00	10 ± 0.00
Total Alkalinity (mg/L CaCO_3_)	110 ± 0.01	120 ± 0.01	120 ± 0.01	110 ± 0.01	110 ± 0.01	120 ± 0.01
Tannic acid (TA) (µM)	1.51 ± 0.00	1.13 ± 0.00	0.90 ± 0.00	1.34 ± 0.00	1.09 ± 0.01	1.03 ± 0.01
Gallic acid (GA) (µM)	0.83 ± 0.01	0.92 ± 0.00	0.50 ± 0.01	0.55 ± 0.00	0.85 ± 0.01	0.39 ± 0.00
Dissolved ammonia (µM)	2.40 ± 0.00	1.95 ± 0.00	1.95 ± 0.00	2.40 ± 0.00	2.50 ± 0.00	2.56 ± 0.00
Dissolved nitrate (µM)	124.42 ± 0.02	143.46 ± 0.00	102.08 ± 0.00	132.60 ± 0.02	173.62 ± 0.01	98.58 ± 0.04
Dissolved *o-*phosphate (µM)	2.63 ± 0.00	5.60 ± 0.00	7.32 ± 0.00	4.23 ± 0.00	4.36 ± 0.00	3.99 ± 0.00
Dissolved reactive silicate (µM)	43.26 ± 0.02	50.18 ± 0.03	41.21 ± 0.05	58.67 ± 0.06	75.3 ± 0.00	60.70 ± 0.00
Dissolved organic carbon (DOC) (mg/L)	59.2	31.6	90.4	21.6	100.4	28.8

**Table 2:** Metal and metalloid ions concentration from surface water of four IMA (IMA_C1, IMA_C3, IMA_DB1, and IMA_DB4) and two non-IMA (C6_NM and DB5_NM) coastal aquaculture ponds located in North 24 Parganas and South 24 Parganas districts, West Bengal, India.

Metal/Metalloid ions concentration (ppm)	IMA_C1	IMA_C3	C6_NM	IMA_DB1	IMA_DB4	DB5_NM
^23^Na	37,290.00	43,680.00	42,090.00	16,056.00	15,831.00	40,560.00
^24^Mg	466.2	551.7	547.5	166.98	175.65	421.2
^31^P	0.11	0.01	0.08	0.00	0.00	0.46
^44^Ca	350.7	356.1	405.3	98.16	145.17	265.02
^52^Cr	0.01	0.01	0.01	0.01	0.01	0.00
^55^Mn	0.23	0.22	0.05	0.02	1.09	0.28
^56^Fe	1.36	3.00	1.24	0.87	8.12	1.05
^59^Co	0.00	0.00	0.00	0.00	0.00	0.00
^60^Ni	0.01	0.01	0.01	0.00	0.01	0.00
^65^Cu	0.02	0.01	0.02	0.02	0.02	0.02
^66^Zn	0.16	0.24	0.07	0.20	0.08	0.23
^75^As	0.01	0.00	0.01	0.00	0.03	0.08
^111^Cd	0.00	0.00	0.00	0.00	0.00	0.00
^208^Pb	0.01	0.01	0.05	0.01	0.00	0.00

Metal–metalloid ion profiles further highlighted the stabilizing effect of mangrove integration. IMA ponds exhibited lower concentrations of Fe, Mn and Na, suggesting reduced ionic stress and a more stable geochemical regime, whereas non-IMA ponds showed elevated metal loads linked to limited mobilization of ions (Table 2). These patterns underscore the buffering capacity of mangrove-linked systems, where natural filtration, litter decomposition, and microbial transformation collectively sustain balanced nutrient stoichiometry, resulting in improved water quality and overall ecosystem resilience in IMA ponds.

**Fig. 1:**
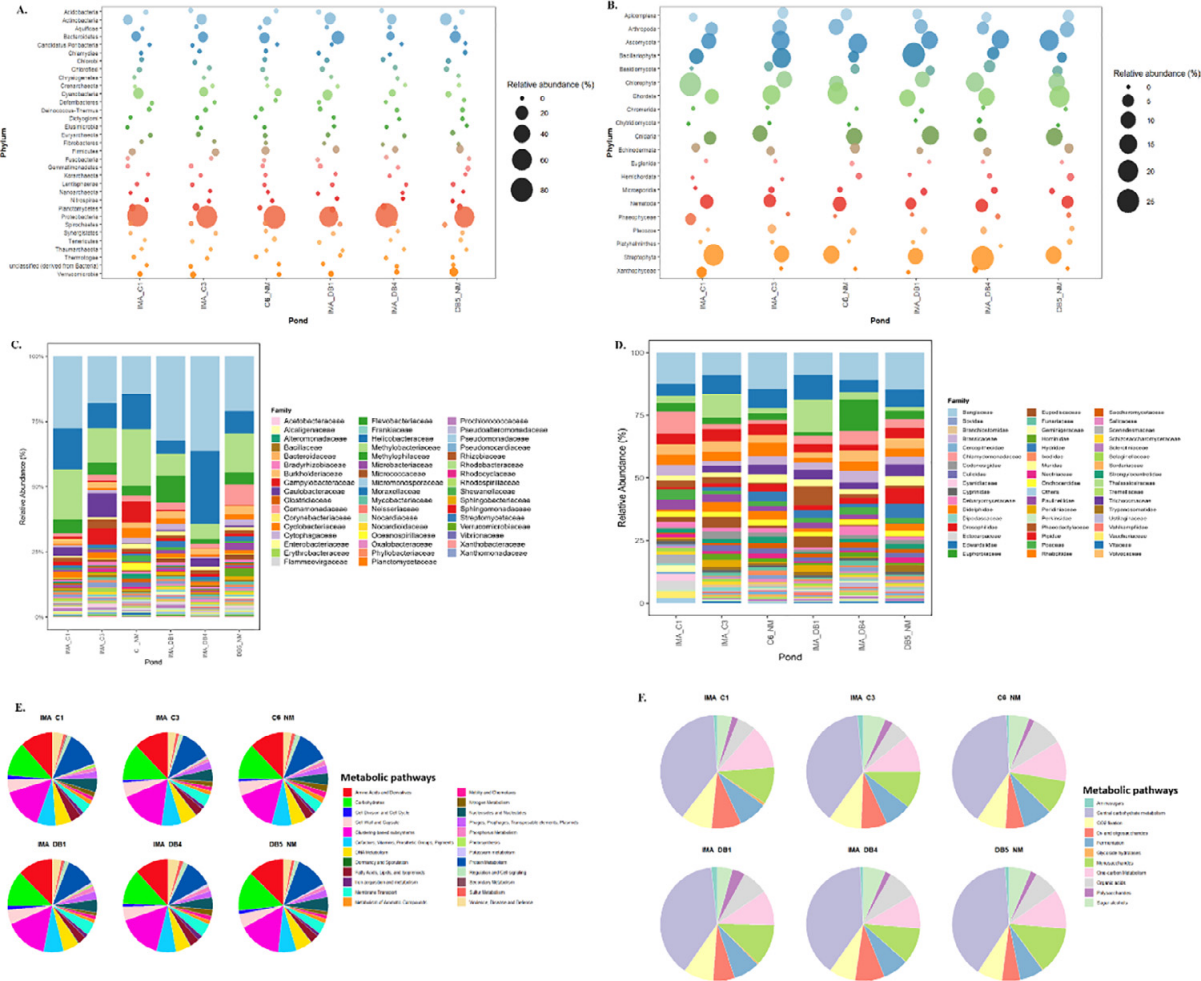
Representing relative abundance of prokaryotic and eukaryotic communities at the phylum (A, B) and family (C, D) levels and their major metabolic functional pathways (E, F) in IMA and non-IMA aquaculture ponds.

This dataset also elucidates the structural and functional potential of microbial communities that are central to biogeochemical cycling in brackish water aquaculture systems. Around 320 MB of eDNA data were generated from the ponds IMA_C1, IMA_C3, C6_NM, IMA_DB1, IMA_DB4, and DB5_NM. The raw sequence data are publicly accessible on server in the NCBI repository: https://www.ncbi.nlm.nih.gov/sra/PRJNA1321950. Taxonomic profiling of the bacterioplankton community at the phylum level revealed five dominant groups (>10 % of the total community): Actinobacteria, Bacteroidetes, Cyanobacteria, Firmicutes, and Proteobacteria. Among these, Proteobacteria constituted >80 % of the total abundance in the IMA ponds, while Actinobacteria and Cyanobacteria each accounted for over 20 % (Fig. 1A). Eukaryotic communities were primarily composed of Bacillariophyta, Chlorophyta, Nematoda, Cnidaria, and Xanthophyceae, each contributing between 20 % and 30 % of the total abundance (Fig. 1B).

At the family level, dominant bacterioplankton included Pseudomonadaceae, Campylobacteraceae, Moraxellaceae, and Methylobacteriaceae, which together accounted for approximately 25 % of the total abundance across all ponds. Helicobacteraceae showed the highest relative abundance in the IMA_DB4 pond, representing nearly 25 % of the community, while Rhodobacteraceae was also prominent, comprising around 15 % of the total abundance (Fig. 1C). In eukaryotes, dominant families included Bangiaceae, Thalassiosiraceae, Trichocomaceae and Volvocaceae (Fig. 1D).

Functional annotation of both prokaryotic and eukaryotic communities revealed genes associated with diverse metabolic processes, including amino acid, carbohydrate, monosaccharide, sugar alcohol, iron acquisition, nitrogen, phosphorus, sulphur, potassium and aromatic compound metabolism. Of these, approximately 45 % of the genes were linked to carbohydrate, amino acid, and aromatic compound metabolism [Fig. 1 (E & F)]. Notably, IMA ponds exhibited higher microbial richness (8 phyla and 42 families) compared to non-IMA ponds (6 phyla and 29 families), along with a greater representation of genes related to nitrogen, sulphur, aromatic compound metabolism and carbon turnover.

These functional attributes indicate enhanced ecological resilience and a more balanced nutrient processing capacity under the influence of integrated mangrove systems. Overall, the findings highlight that mangrove litter-driven microbe interactions play a pivotal role in maintaining the ecological stability of ecosystem by supporting efficient nutrient transport, organic matter degradation, and improved ecosystem health within IMA ponds.

## 4 Experimental Design, Materials and Methods

### 4.1 Study site

This study was performed in the surrounding region of the Indian Sundarbans, covering the North 24 Parganas and South 24 Parganas districts of West Bengal, India, specifically in the Minakha Block (Chaital; 22°29′59.8 N 088°47′56.2 E) and Kultali subdivision (Madhabpur; 22°03′19.8 N 088°34′13.6 E). These districts are extensively used for brackish-water aquaculture, primarily farming tiger shrimp (*Penaeus monodon*) and freshwater giant prawn (*Macrobrachium rosenbergii)*. This region represents a unique estuarine ecosystem formed by the convergence of freshwater inflows from the Bidyadhari and Matla Rivers with saline tidal waters from the northeastern Bay of Bengal, creating ideal conditions for brackish-water aquaculture [4,5]. Natural mangrove patches further enhance biodiversity by providing breeding grounds and sustaining diverse coastal bioresources, thereby strengthening ecosystem resilience.

Total active aquaculture area is ∼ 45,000 ha in North 24 Parganas and ∼ 55,000 ha in South 24 Parganas. Of this, approximately 10 % of the geographical area of North 24 Parganas and 90 % of South 24 Parganas accounts for brackish water aquaculture, as highlighted in the NFDB and MPEDA 2021, 2022 and 2023 reports. In these regions, aquaculture practitioners often adopt sustainable aquaculture practices such as Integrated Mangrove Aquaculture (IMA) and Sustainable Aquaculture in Mangrove Ecosystem Fisheries (SAIME) [6]. These approaches involve planting mangroves along pond peripheries and establishing mangrove-covered islands within ponds, interconnected through canal channels. Such designs mimic natural estuarine ecosystems, promoting nutrient cycling from senescent mangrove leaf litter, improving water quality and providing enhanced habitats for shrimp and other bioresources growth [[8], [9], [10]].

Mangrove leaf litter contributes organic matter to detrital food webs, supporting the growth of beneficial microbial communities and, in turn, enhancing shrimp production. Beyond improving yields, IMA and SAIME practices serve as natural buffers against extreme meteorological events and enhance blue carbon sequestration. Together, these sustainable approaches promote long-term ecological balance while securing better livelihood opportunities for the fisherfolk communities with improved bioresource yields.

### 4.2 Sampling

Sampling was done in two IMA ponds (IMA_C1, IMA_C3) and a non-IMA pond (C6_NM) at Chaital, and two IMA ponds (IMA_DB1, IMA_DB4) and a non-IMA pond (DB5_NM) at Madhabpur during the monsoon season of October 2022. From each sampling pond, 1 L of surface water was collected and immediately fixed with buffered 4 % formalin (Merck, Germany) to estimate dissolved nutrients (ammonia, nitrate, *o*-phosphate, and reactive silicate) and total alkalinity concentrations following published methodologies [11]. In addition, 1 L of surface water was collected from each pond and immediately fixed with molecular-grade ethanol (Merck, Germany) for environmental DNA (eDNA) extraction. Unfixed, filtered surface water samples were collected for dissolved organic carbon (DOC) and phenolics (tannic acid [TA] and gallic acid [GA]) estimation, while filtered samples fixed with 2 % Suprapur HNO₃ (Merck, Germany) were collected for metal and metalloid estimation. The collected samples were ice-packed after collection and immediately transported to the laboratory for downstream analysis.

### 4.3 Measurement of *in-situ* environmental parameters

At the time of sampling, *in-situ* environmental parameters were measured. Air temperature (AT, °C) and surface water temperature (SWT, °C) were recorded using a digital thermometer (Digi-sense RTD meter 20,250–95, single-input thermometer with NIST-traceable calibration). Dissolved oxygen (DO, mg/L) was measured with a hand-held probe (Hanna Instruments HI98193, EU, with temperature sensor), while pH was determined using a pH probe (Hanna Instruments HI98190, EU, with temperature sensor). Salinity, total dissolved solids (TDS, ppm), and electrical conductivity (EC, µS/cm) were measured with a multiparameter probe (HI98192 EC/TDS/Resistivity/Salinity Meter, with temperature sensor, Hanna Instruments, EU). Water transparency (Secchi depth, cm) was estimated using a Secchi disc (LaMotte, France). All instruments were calibrated both in the laboratory and in the field, following the manufacturers’ protocols. Measurements were taken in triplicate, and contamination was minimized by cleaning instruments with milli-Q water and wearing nitrile gloves during sampling.

### 4.4 Estimation of tannic and gallic acids, dissolved nutrients and total alkalinity

Surface water samples were filtered through 0.22 μm pore size, 25 mm diameter nitrocellulose syringe filters (Whatman Uniflo; UK). Tannic acid (TA) and gallic acid (GA) concentrations were quantified using Folin phenol reagent and UV–Vis spectrophotometer (U2900, Hitachi, Japan) following established protocols [8,12]. Dissolved nutrients, including ammonia, nitrate, *o*-phosphate, and reactive silicate, were measured by UV–Vis spectrophotometer using standard colorimetric methods [11]. Total alkalinity was determined by titration with 0.02 N H₂SO₄ against Bromocresol Green (BCG) indicator. All analyses were conducted in triplicate.

### 4.5 Estimation of dissolved organic carbon (DOC)

Surface water samples were filtered through 0.22 μm pore size, 25 mm diameter nitrocellulose syringe filters (Whatman-uniflow, UK) and DOC was measured by thermos catalytic combustion at 850 °C using a multi N/C 2100S analyzer (Analytik Jena AG, Germany). All analyses were performed in triplicate, with the coefficient of variation (CV) maintained below 4 % for each sample run [13].

### 4.6 Metal and metalloid analyses using inductively coupled plasma masss pectrometry (ICP-MS)

Surface water samples from Chaital (IMA_C1, IMA_C3, and C6_NM) and Madhabpur (IMA_DB1, IMA_DB4, and DB5_NM) were analyzed for metals and metalloids. Samples were filtered through 0.22 μm, 25 mm nitrocellulose syringe filters (Whatman-Uniflo, United Kingdom) and preserved on-site with 2 % Suprapur nitric acid (Merck, Germany) for further digestion to be carried out at 60 °C. Concentrations were determined using an inductively coupled plasma mass spectrometer (ICP-MS; iCAP RQplus, Thermo Scientific, United States of America) with FINAR-92 multi-element standards (Christiansburg, USA). Calibration was carried out using Certified Reference Materials (CRMs; BCR 617 and BCR 610, EVISA, EU). Instrument parameters included plasma power of 1549 W, nebulizer gas flow of 1.045 L/min with a back pressure of 3.20 bar, auxiliary gas flow of 0.80 L/min, coolant gas flow of 13.9 L/min, CCT gas flow of 4.43 mL/min and a peristaltic pump speed of 40 rpm.

### 4.7 Environmental DNA (eDNA) extractionand nanoporesequencing

Environmental DNA (eDNA) was extracted from surface water collected from each pond following published protocol [14,15]. Libraries were prepared from 200 ng of purified eDNA using the Ligation Sequencing Kit (SQK-LSK109) and Native Barcoding Kit (EXP-PCR096) (Oxford Nanopore Technologies, UK). End-repair, dA-tailing and adapter ligation were performed following the manufacturer’s workflow, with barcode attachment for multiplexing and purification using AMPure XP beads. Then pooled library was quantified using a Qubit 4.0 fluorometer and loaded onto a MinION R9.4.1 (FLO-MIN106) flow cell for a 48-hrs sequencing run. Base-calling and demultiplexing of raw reads FASTA files were performed using Guppy v2.3.4 (Oxford Nanopore Technologies; available at https://community.nanoporetech.com), generating high-quality long-read data in FASTQ format for downstream taxonomic and functional annotation.

### 4.8 Data analysis

Raw sequencing reads in FASTQ format were uploaded to the MG-RAST pipeline [16] for analysis. Quality control involved removing low-quality and duplicate reads, followed by adapter and barcode trimming. High-quality datasets were normalized prior to downstream analyses. Taxonomic profiling was carried out using clustering and similarity-based annotation against the SILVA v138 database, providing accurate classification of microbial communities. In parallel, functional annotation was performed using MG-RAST against SEED subsystems, KEGG Orthology (KO), and COG databases, allowing the identification of microbial functional roles related to metabolism, nutrient cycling and ecological interactions.

## Limitations

Not applicable

## Ethics Statement

The work described above did not involve human or animal subjects; therefore, no regulatory compliance guidelines were applicable.

## CRediT Author Statement

Yash undertook field sampling, performed the experimental work and drafted the initial version of the manuscript. A.G. contributed to the conceptualization, validation of analysis and edited the manuscript. A.D. contributed to the conceptualization, undertook funding acquisition and edited the manuscript. M.S. contributed to the field sampling and edited the manuscript. N.B. contributed to the field sampling and edited the manuscript. S.S. contributed to the field sampling and edited the manuscript. P.B. contributed to conceptualization, undertook funding acquisition and project administration, participated in field work, supervised the work, drafted and edited the manuscript.

## Data Availability

NCBIUnderstanding the microbial community structure of Integrated Mangrove Aquaculture Pond (IMA) (Original data)
